# Comments on Behavior of Healthcare Workers After Injuries From Sharp Instruments

**DOI:** 10.5812/traumamon.17040

**Published:** 2014-01-25

**Authors:** Seyed Moayed Alavian

**Affiliations:** 1 Baqiyatallah Research Center for Gastroenterology and Liver Diseases, Baqiyatallah University of Medical Sciences, Tehran, IR Iran

**Keywords:** Health Personnel, Prevention and Control, Needlestick Injuries, Hepatitis B, Hepatitis C

*Dear Editor,*

I read the recently published article by Adib-Hajbaghery et al. (1) in your journal with interest. Healthcare workers (HWCs) are at high risk of infection with hepatitis B virus (HBV) and hepatitis C virus (HCV) transmitted through blood and infected fluids; the infected staff can transfer these viruses to uninfected patients while providing services (2). Adib-Hajbaghery et al. (1) reported that around 90% of the enrolled cases had received hepatitis B vaccination. The authors did not present any data regarding the postvaccination anti-HBs antibody titres. According to the standard precautions for infection control, all HWCs susceptible to the infection should be identified and immunized to reduce the morbidity rate; thus the evaluation of anti-HBs antibody level is mandatory. HWCs, particularly those working in the emergency departments, operating rooms and hemodialysis centers are considered as high-risk groups (2). In addition to HBV infection, the HWCs are occupationally at the risk of HCV infection (3). Unfortunately, there is no passive or active prevention for HCV infections and HCWs should be more cautious and apply the standard of health precautions at work. Adib-Hajbaghery et al. (1) showed that more than 50% of the people injured with sharp instruments did not follow the standard precautions. Compression and washing the area with soap and water cannot prevent HBV and HCV infections. In their study group, 38.3% had a history of needle-stick injury, or injuries with sharps within the last six months. A higher rate of exposure was reported in the study on HCWs by Shokuhi et al. (4), occupational exposure to blood and body fluids of patients was reported in 53.4% of cases (4). In the study conducted by Shokuhi et al., 25.8% of HWCs with a history of needle-stick injury or mucosal exposure to HBs Ag (+) patients' blood or body fluid, received hepatitis B immunoglobulin (HBIG) within the first 72 hours after exposure. History of vaccination, and assurance about the effective serum antibody titer were the most common reasons that the individuals did not receive HBIG (56.5%) (4). Finally, I would like to emphasize on the necessity to educate HWCs especially the operating room nurses and midwives who are at the high risk of needle-stick, and sharps injuries. More education about the prevention of repeated injuries is critical for this high-risk group (2, 4). There is a need for further research to investigate why many HWCs do not take prophylactic and essential actions after needle stick injury or mucosal exposure to body fluids of infected patients, and why the health systems do not appreciate the importance of this issue in HWCs (4).
